# Validation Study of Adherence to Daily Multiple Micronutrient Supplementation by Palestine Refugee Women Attending United nations Relief and Works Agency for Palestine Refugees in the Near East Health Clinics in Jordan

**DOI:** 10.1016/j.cdnut.2025.107588

**Published:** 2025-10-30

**Authors:** Masako Horino, Luai Al-Khatib, Rami Habash, Tamara Al-Rahahleh, Akihiro Seita, Saifuddin Ahmed, Klaus Kraemer, Kristen M Hurley, Keith P West

**Affiliations:** 1 United Nations Relief and Works Agency for Palestine Refugees in the Near East (UNRWA), Health Department, Amman, Jordan; 2 Center of Human Nutrition, Department of International Health, Johns Hopkins Bloomberg School of Public Health, Baltimore, MD, United States; 3 Department of Family and Reproductive Health, Johns Hopkins Bloomberg School of Public Health, Baltimore, MD, United States; 4 Sight and Life Foundation, Basel, Switzerland; 5 Vitamin Angel Alliance, Goleta, CA, United States

**Keywords:** validation, adherence, antenatal care, multiple micronutrient supplementation, implementation research, Palestine refugees

## Abstract

**Background:**

Multiple micronutrient supplementation (MMS) is a new standard of antenatal care emerging in lower-income countries. Assessing adherence, a key implementation outcome, requires validation.

**Objectives:**

Validate antenatal adherence to daily MMS based on recalled tablet days missed during follow-up intervals in UNRWA clinics serving Palestine refugees in Jordan.

**Methods:**

This study was conducted during an implementation trial of antenatal MMS versus standard-of-care iron-folic acid supplementation. In 13 clinics, at registration, pregnant women received daily MMS in 180-count bottles weighing 111 g (bottle with desiccant = 26 g, tablet = 0.47 g). At seven follow-up visits, returning bottles were weighed to nearest gram on digital scales, and recalled tablet days missed were recorded. Tablets removed from bottles, estimated from bottle weight decrements, were regressed on tablets taken, obtained by subtracting tablet days missed from interval lengths. Similar analyses were conducted for cumulative intervals since registration.

**Results:**

Among 9754 registered women, 7608 (78%), 6,208 (63.6%), 4,833 (49.5%), 3,409 (34.9%), 2159 (22.1%), 1063 (10.9%), and 384 (3.9%) completed the first-to-seventh follow-up visits, respectively, with a decline mainly due to censoring at close-out. Approximately 25% of women failed to return bottles, whose tablet intakes were modestly lower than those of bottle-returnees. Notwithstanding, 75 to 84% in both groups recalled missing <2 d and <5.3% missing >30 d. Interval-specific regression slopes (β_1_) were 0.88–0.78 [95% confidence intervals (CIs) within +0.06 for first 5 intervals], reflecting a ∼20% over-reporting by recall, with r = 0.77–0.58, and root mean square errors (RMSE) of 10–12 tablets, reflecting model error in prediction, normalized to 7%–10% of interval ranges. Cumulative models yielded β_1_ = 0.83–0.77 with similarly narrow 95% CIs, r = 0.79–0.64, and RMSE = 12–24 tablets, normalized to 7%–13% of range.

**Conclusions:**

Histories of recalled tablet days missed provide a reliable and reasonably valid estimate of tablet disappearance from MMS bottles. Further research is warranted to correct a consistently reported ∼20% average overestimate of tablet intake.

Micronutrient deficiencies (MNDs) present a global burden affecting an estimated 1 billion women of reproductive age in low- and middle-income countries [1]. Pregnant women are susceptible to MNDs as maternal requirements for essential micronutrients are increased to accommodate metabolic demands of growing fetal, placental, and maternal tissues [2]. Consequences of micronutrient deficiencies during pregnancy include maternal anemia [[2], [3], [4]] and increased risks of preterm, small-for-gestational age, low weight, and still births [2,5].

In Palestine refugee communities in the Middle East, coexisting gestational deficiencies of vitamins A, D, E, and B-complex, iron, zinc, and iodine have been reported [[6], [7], [8], [9], [10]]. Maternal anemia (Hb <11 g/dL) of nutritional origin has been reported in Jordan [11], and early and mid-pregnancy anemia rates of 26% and 54%, respectively, have been reported from clinics operated by the United Nations Relief and Works Agency for Palestine Refugee in Near East (UNRWA) in the region [12].

Since the 1990s, UNRWA has prescribed iron and folic acid (IFA) supplements to prevent anemia among refugee pregnant women in Jordan, Lebanon, Syria, the West Bank and the Gaza Strip [13], guided by a standing Technical Instruction (policy) to provide 1 tablet of folic acid (FA) daily in the first trimester, followed by IFA, prescribed at variable weekly frequencies per clinical judgment, thereafter throughout pregnancy [14]. Both FA and IFA tablets are provided in 10-count blister packs.

Given evidence supporting improved pregnancy outcomes from large, randomized trials of MMS versus IFA [15,16], the WHO issued in 2020 an updated guideline on antenatal MMS, stating the intervention is recommended for use “in the context of rigorous research” [17], including implementation research where MMS delivery is being considered. To address the burden of MNDs among Palestine refugee gravidae, since 2020 UNRWA has been planning and testing the delivery and use of the United Nations International Multiple Micronutrient Approved Preparation (UNIMMAP) formulated supplement [18,19], providing 15 essential micronutrients at Recommended Dietary Allowances (RDA) specified for pregnancy [20], and as found on the WHO Essential Medicines List (Supplementary Table 1) [21]. The goal of the Agency’s implementation research strategy has been to inform and guide the eventual scale-up of daily MMS to replace an existing FA/IFA regimen that has served as a standard of prophylactic antenatal care. Following 2 y of planning and preparation, the Agency conducted a 6-mo pilot phase in 2 clinics in Jordan [22], followed by a 10-mo system-wide trial in Jordan to compare a range of implementation outcomes of MMS versus FA/IFA delivery, which was completed in December 2023.

A key implementation outcome is the extent to which pregnant women adhere to a prescribed daily prophylactic regimen. Although technical guides have appeared to guide implementation research on MMS [23,24], data remain sparse on validity of methods to assess MMS adherence during routine antenatal visits. In this article, we report a prospective approach to validate a maternal history of MMS consumed from a bottle by asking about days missed taking the supplement.

## Methods

From March 1 to December 31, 2023, an evaluation was conducted by UNRWA in its 25 antenatal clinics serving Palestine refugees in Jordan. In this study, prophylactic delivery and use of daily MMS was compared with a standard-of-care regimen of prescribing daily supplemental FA (0.4 mg) in the first trimester, followed by prescriptions of 2, 3 or 7 tablets of ferrous fumarate (100 mg elemental iron with 0.35 mg FA) weekly, per clinical judgment, starting in the second trimester [14]. The UNIMMAP MMS tablet, with RDAs of 15 essential micronutrients [20] at dosages referenced on the WHO Essential Medicines List (Supplementary Table 1) [21], is US Pharmacopoeia- and halal-certified [18]. Supplements were supplied *gratis* to the Agency in 180-count, opaque, child-proof, desiccant-protected plastic bottles by the Kirk Humanitarian Foundation (Salt Lake City). The supplement was approved for in-country use by the Jordan Food and Drug Administration in 2022 [22].

### Piloting of MMS program

Prior to launching a formal evaluation, MMS was piloted for 6 mo (September 1, 2022 to February 29, 2023) in 2 UNRWA clinics, located in Amman New Camp and Marka Camp [22], selected for their large client populations, central locations, and excellent relationships in their respective communities. This phase allowed the Agency to orient staff to the new supplement, assess workflow, develop study procedures, training materials, midwife data collection methods, and recipient education messages, and revise programming of the Agency’s eHealth system to accommodate several new variables. Given the 180-ct plastic bottle in which MMS would be delivered, a parallel goal of this phase was to develop an adherence assessment protocol that could be followed, measuring bottle weights under usual busy clinic conditions. Also, during the pilot phase, 5 full bottles with caps and desiccant were initially weighed and reweighed after removing 1 tablet until empty, leading to a calculated mean (SD) tablet weight of 0.47 (0.003) g, used as the value to estimate MMS tablets removed from weighed bottles. An empty bottle with its cap and desiccant was determined to weigh 26 g.

**FIGURE 1:**
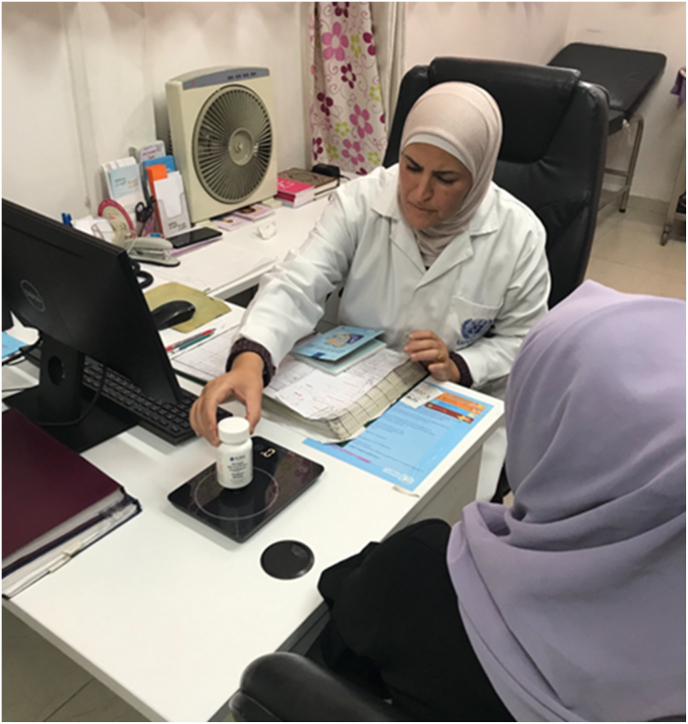
Midwife weighing a returned multiple micronutrient supplementation (MMS) bottle on a digital scale during a follow-up antenatal visit, UNRWA Health Center, Marka Camp, Jordan, 2023 (photo with permission). UNRWA, United Nations Relief and Works Agency for Palestine Refugees in the Near East.

In exploring tablet intake histories during the pilot phase, midwives noted that mothers more readily recalled the number of days missed than days having taken a supplement since a previous visit. Thus, during the trial in which there were 13 MMS-assigned clinics, women were asked to return with their bottle at each follow-up visit. Recipients were asked about prescribed days missed, and, independent of questions asked, midwives weighed bottles on small, calibrated digital kitchen scale placed in each midwife’s desk (Figure 1). Bottle weight was entered to the nearest 1 g into the mother’s eHealth record. Data on recalled missed days, coupled with interval lengths in days obtained from known clinic visit dates, provided a basis for calculating interval-specific and cumulative percent supplement adherence at each visit.

As FA and IFA tablets were routinely dispensed in variable numbers of 10-tablet blister packs at all antenatal care visits, we could not identify a comparable, practical methodology to reliably and independently validate reported adherence to prescribed use of FA and IFA tablets under clinic conditions. Consequently, in the formal evaluation [25], the question to recall days missed of prescribed FA and IFA supplement use was not validated or reported here.

### Staff training

In November 2022, 4 sessions of 1-d in-person training were provided by UNRWA investigators and Jordan Field Office health officers at UNRWA Headquarters in Amman, Jordan, to midwives, nurses, physicians, and pharmacists assigned to MMS- and FA/IFA-assigned clinics. Training curriculum included the study design, respective supplement prescriptive, issuance, and documentation procedures, recording responses on supplement use and complaints of side effects, patient instructional materials, and eHealth data entry procedures. Subsequently, 4 assistants with bachelor's degrees, who were previously oriented to the trial and trained on eHealth data collection and entry procedures periodically visited clinics to discuss, address questions, and refresh knowledge of study procedures.

### Clinic assignment to supplement regimens

For the formal evaluation, UNRWA’s 25 Jordanian clinics were assigned to MMS or to continue dispensing the Agency’s standard of care FA/IFA regimen. Both pilot clinics continued providing MMS and remained in the study, whereas the remaining 23 clinics were ordered by size (annual antenatal visits), stratified by location in or outside of a camp within each of 4 governates and, following a random start systematically assigned to deliver MMS (n = 11) or FA/IFA (n = 12) supplement regimens [[25], [26], [27]]. Subsequently, for pragmatic reasons, the UNRWA Health Department reassigned 2 clinics initially randomized to FA/IFA to MMS and 2 clinics initially randomized to MMS to FA/IFA supplementation. These steps led to 13 and 12 clinics in total being assigned to MMS and IFA, respectively, for the implementation trial.

### MMS adherence validation

The adherence validation study was restricted to women whose hemoglobin (Hb) at registration, routinely measured as part of a complete blood count, was >10 g/L, the cutoff above which women were prescribed daily prophylaxis, and below which therapeutic IFA plus MMS was prescribed, and thus excluded from this analysis. During the trial, eligible women were issued one 180-count bottle of MMS by midwives, and advised to take 1 tablet daily, close the screw top lid tightly after use, keep desiccant in the bottle, not share supplements, and bring back the bottle at each follow-up visit. A sticker indicating date of bottle issuance with a printed reminder to bring back the bottle at each clinic visit was placed on the cover page of a standard maternal and child health booklet routinely provided to all gravidae at registration (seen on desk in Figure 1).

At all follow-up visits, midwives asked women “since the last visit, on (*day, month, year*), how many days did you miss taking “*vitamin*” tablets received from UNRWA?” Midwives weighed the returned MMS bottle with cap to the nearest 1 g on the same model digital kitchen scale (Vont Innovations) (Figure 1). Responses were entered into the eHealth system. “Don’t know” and “no bottle” responses were permitted if a bottle was not weighed or brought back, respectively, at a visit.

### Validation study sample size

Based on previous health system records, ∼20,000 Palestine refugee pregnant women were expected to register for care in UNRWA’s 25 Jordan clinics in 2023, or ∼16,000 over a 10-mo study period, comparably distributed each month from March 1, 2023, to December 31, 2023, when pregnant women receiving care were censored from the study, irrespective of month of registration or completed follow-up visits. With 13 MMS-assigned clinics, some of which were larger in size, we expected 60%–65%, or ∼10,000 registrants for the adherence evaluation, or ∼1000 per month, with earlier registrants contributing more follow-up visits, declining to none among December registrants. With this imposed design, and likely follow-up intervals of 30–40 d, we estimated roughly at least half, or >5000 MMS-recipients, would contribute 3–4 follow-up visits. With little data on MMS-related implementation outcomes of interest guiding hypothesis-driven sample size estimation, expected numbers were pragmatically considered sufficient to estimate and conduct a substudy to validate adherence.

### Data abstraction

Data for this study were entered by midwives into the UNRWA Health Department’s eHealth database. Except for clinic name and relevant dates, all study records were deidentified and recoded prior to release to investigators for analysis. Output data included clinic name, recipient age, parity, education, and residence within or outside a camp, dates of registration, last menstrual period, MMS bottle receipt and follow-up visits, recalled days missed taking MMS during each previous interval, and MMS bottle weight in grams at each visit.

### Statistical analysis

Analysis was restricted to women who registered for care at any trimester between March 1 and December 31, 2023, had an initial Hb ≥ 10 g/dL, were issued a single 180-count bottle of MMS at registration, and may have returned for ≤ 7 follow-up antenatal visits. At follow-up visits, recalled days missed during each interval were summarized as frequency distributions stratified as having missed <2, 3–4, 5–9, 10–29, and >30 (and >10) days per interval, excluding “don’t know” responses, and treated as continuous distributions for later regression analyses. Recipients were stratified, and characteristics and adherence were analyzed with respect to compliance in returning bottles at each follow-up visit. Bottle weight distributions were examined, with values excluded if they were recorded outside a plausible range of 26 g (empty bottle) to 111 g (full bottle). Variables related to adherence assessment were summarized by frequency distributions, contingency tables, and medians with IQRs for ordered variables (e.g., weeks of gestation, days per follow-up interval, and days since registration or a previous visit). Pearson’s ꭓ^2^ was employed to examine differences in categorical variables.

Among women with both recalled MMS days missed and bottle weight data, we calculated the following adherence validity-related intermediates:

(a) Days since registration = date of current follow-up visit – date of pregnancy registration;
(b) Follow-up interval (days) = date of current follow-up visit – date of previous visit;
(c) Tablets missed per interval = recalled tablet days missed since previous visit;
(d) Tablets missed, cumulatively = sum of all preceding interval-specific recalled tablet days missed;
(e) Tablets recalled taken during a specific interval = (b) – (c)
(f) Cumulative tablets recalled taken since registration = (a) – (d)
(g) Tablets left in bottle based on recalled tablet days missed = 180 – (d)
(h) Tablets removed from bottle during an interval = [bottle weight (g) at previous visit – bottle weight (g) at specific follow-up visit] / 0.47 g
(i) Cumulative tablets removed from bottle = [111 g – bottle weight (g) at specific follow-up visit] / 0.47 g

To evaluate the validity of our recalled missed days approach to assessing MMS adherence, we fit simple linear regression models to examine the linearity and strength of association between tablets removed and taken from bottles, by regressing estimated tablets removed during a specific interval (equation h) or cumulatively (equation i) based on decrements in bottle weight (y-variables), on tablets recalled taken from bottles in each specific (equation e) or cumulative (equation f) interval (x-variables). In the case of unbiased reporting, the slope of each interval-specific and cumulative model is expected to be equal to one (β_1_ = 1). Ninety-five percent confidence intervals (95% CI) for β_1_ were constructed from standard errors. Root mean square errors (RMSE) and RMSEs normalized to each range (NRMSE) for tablets removed, estimated from bottle weight decrements, were calculated to quantify imprecision in interval-specific and cumulative distribution models [28]. We further superimposed linear regression lines on scatterplots to visually inspect joint distributions.

We considered a *P* value <0.05 applied to comparing discreet and continuous distributions by Chi-squared test and Student’s t-test, as appropriate, to be statistically significant. Missing values were documented in footnotes to tables and figures. All analyses were done using Stata, version 18 (StataCorp LLC).

### Ethical review and approval

The study protocol, which included assessment of adherence and its validation, was classified as a program evaluation of health services and exempted per UNRWA policy (UNRWA. Executive office instruction No. 04. Terms of references of the research review board and ethical standards in research involving individuals, Amman, Jordan. 12 March 2019) from review by the Agency’s Research Review Board in Amman, Jordan. The protocol was separately reviewed and approved as nonhuman subjects research/public health practice by the Johns Hopkins Bloomberg School of Public Health (JHSPH) Institutional Review Board in Baltimore, Maryland, United States.

## Results

A total of 10,402 pregnant women registered for antenatal care in the 13 MMS-assigned clinics, of whom 648 (6.2%) had an initial Hb<10 g/dL, were started on an anemia treatment protocol, and excluded from this analysis (data not shown). The remaining 9754 (93.8%) registrants had an initial Hb≥10 g/dL, were placed on MMS prophylaxis and eligible for this study, of whom 6,617 (68.1%), 2,826 (29.1%), and 271 (2.8%) registered in the first (≤12 wk of gestation), second (13–27 wk), and third (≥ 28 wk) trimesters, respectively (Table 1). Approximately 10% of the total sample registered each month (except for lower and higher percentages in Ramadan and post-Ramadan months of April and May, respectively) (Supplementary Table 2). Across all registrants, 57.9% were 20–29 y of age, 45.6% had 1–10 y of education, 23.2% lived within formal refugee camp boundaries, and 25.2% were primiparous, although third trimester registrants were more likely to be multiparous. Overall, 78% of registrants completed the first follow-up, declining to 63.6%, 49.5%, 34.9%, 22.1%, 10.9%, and 3.9% completing the subsequent 6 sequential follow-up visits, respectively (Table 1), the reduction largely reflecting censoring of data on December 31, 2023, regardless of number of visits completed, coupled with fewer visits made by later trimester registrants (Table 1).

**TABLE 1:** Characteristics of Palestine refugee women eligible for MMS prophylaxis^1^ by trimester of registration, 13 MMS-assigned UNRWA clinics, Jordan, March to December 2023.

	First trimester		Second trimester		Third trimester		All trimesters	
	n	%	n	%	n	%	n	%
All eligible women, N (%)	6617^2^	68.1^2^	2826^2^	29.1^2^	271^2^	2.8^2^	9754^2^^,^^3^	100^2^
Age category, years, N (%)	6617	100	2826	100	271	100	9754^3^	100
15–19	550	8.3	210	7.4	13	4.8	777	8.0
20–29	3834	57.9	1635	57.9	154	56.8	5644	57.9^2^
30–39	1979	29.9	859	30.4	93	34.3	2945	30.2
40–49	254	3.8	122	4.3	11	4.1	388	4.0
Education^4^, N, %	6607	99.8	2818	99.7	271	100	9736^3^	99.8
Grades 1–10	3038	46.0	1241	44.0	127	46.9	4435	45.6^2^
Grades 11–12	2488	37.7	1022	36.3	95	35.1	3614	37.1
Some college or higher	1081	16.4	555	19.7	49	18.1	1687	17.3
Residence^5^, N, %	6264	94.7	2651	93.8	259	95.6	9213^3^	94.5
Inside camp	1441	23.0	618	23.3	69	26.6	2141	23.2^2^
Outside camp	4823	77.0	2033	76.7	190	73.4	7072	76.8
Parity, N, %	6617	100	2826	100	271	100	9754^3^	100
0	1109	16.8	315	11.1	14	5.2	1443	14.8
1	1657	25.0	707	25.0	88	32.5	2459	25.2^2^
2–4	3214	48.6	1376	48.7	116	42.8	4726	48.5
5 or more	637	9.6	428	15.1	53	19.6	1126	11.5
Antenatal care visits, N, %								
Registration	6617	100	2826	100.0	271	100.0	9754^3^	100.0
First follow-up	5124	77.4	2262	80.0	215	79.3	7608	78.0^2^
Second follow-up	4182	63.2	1879	66.5	145	53.5	6208	63.6^2^
Third follow-up	3301	49.9	1436	50.8	96	35.4	4833	49.5^2^
Fourth follow-up	2493	37.7	889	31.5	27	10.0	3409	34.9^2^
Fifth follow-up	1720	26.0	434	15.4	5	1.8	2159	22.1^2^
Sixth follow-up	891	13.5	171	6.1	5	1.8	1063	10.9^2^
Seventh follow-up or more	335	5.1	49	1.7	1	0.4	384	3.9^2^

1 Among 10,402 pregnant women who registered in MMS-assigned clinic during the 10-mo implementation trial, 648 had a Hb <10 g/L and referred for treatment, leaving 9754 women in this analysis whose Hb at registration was >10 g/L and thus eligible for daily multiple micronutrient supplements (MMS) prophylaxis.

2 Numbers are those mentioned in Results text.

3 Records for n = 40 of the total of 9754 women in the study were missing trimester of registration, and thus trimester-specific data, which variably affect subset totals within each panel.

4 Records for n = 18 women across trimesters were missing education level.

5 Records for n = 154 women were excluded from residential summary due to reported residence abroad.

Variables related to follow-up visits and intervals, and adherence assessment, including recalled MMS days missed and bottle weights at visits, are summarized in Table 2. Across median intervals of 29–32 d prior to all but the seventh follow-up visit, maternal tablet recall data were available for 92.6%–98.6% of women seen by midwives and thus eligible for adherence assessment. Of these, 75.5%–83.3% reported missing <2 d per interval, whereas 5.3%–7.4% reported having missed taking ≥10 d taking tablets. In-range bottle weight data (26–111 g) were available for 63.4%–76.8% of all eligible adherence assessments, with missing data mostly due to 22.7%–30% of women failing to return with their bottles. Bottle weights shifted downward over time (Table 2). Recall plus weight data were available for 59.6%–72.5% of records through the first 5 follow-up visits, decreasing to 48.4%–38.5% at the sixth and seventh visits, corresponding to censoring patterns.

**TABLE 2:** Summaries of validation-relevant variables collected among all trimester registrants eligible for MMS prophylaxis^1^ followed from first to seventh follow-up visits, 13 UNRWA MMS-assigned clinics, Jordan, March to December 2023.

Variables	Antenatal care follow-up visits (N = 9754 registrants)													
	First follow-up visit		Second follow-up visit		Third follow-up visit		Fourth follow-up visit		Fifth follow-up visit		Sixth follow-up visit		Seventh follow-up visit	
All women, N, % of registrants	7608	78.0	6208	63.6	4833	49.5	3409	34.9	2159	22.1	1063	10.9	384	3.9
Time variables, median (IQR)														
Week of gestation^2^	14^3^	(11–20)^3^	19^3^	(16–25)^3^	24^3^	(20–29)^3^	28^3^	(24–32)^3^	31^3^	(28–34)^3^	33^3^	(31–35)^3^	35^3^	(31–37)^3^
Days per interval	31^3^	(15–41)^3^	32^3^	(28–40)^3^	32^3^	(28–37)^3^	32^3^	(28–35)^3^	31^3^	(24–34)^3^	29^3^	(21–32)^3^	23^3^	(15–31)^3^
Days since registration	31^3^	(15–41)^3^	63.5^3^	(48–79)^3^	96^3^	(78–114)^3^	126^3^	(109–145)^3^	154^3^	(134–170)^3^	173^3^	(151–187)^3^	180^3^	(160–197)^3^
Adherence assessment^4^, N, %	7042	92.6^3^	5925	95.4^3^	4657	96.4^3^	3338	97.9^3^	2096	97.1^3^	1048	98.6^3^	377	98.2^3^
Tablet recall data^5^, N, %^6^	6918	98.2	5803	97.9	4545	97.6	3258	97.6	2043	97.5	1021	97.4	360	95.5
0–2 d missed	5398	78.0^3^	4384	75.5^3^	3459	76.1^3^	2528	77.5^3^	1613	78.9^3^	826	80.9^3^	300	83.3^3^
3–4 d missed	574	8.3	556	9.6	453	10.0	275	8.4	156	7.6	74	7.2	22	6.1
5–9 d missed	473	6.8	447	7.7	325	7.2	239	7.3	143	7.0	46	4.5	19	5.3
10–29 d missed	345	5.0^3^	301	5.2^3^	208	4.6^3^	160	4.9^3^	92	4.5^3^	56	5.5^3^	14	3.9^3^
30+ days missed	128	1.9^3^	115	2.0^3^	100	2.2^3^	56	1.7^3^	39	1.9^3^	19	1.9^3^	5	1.4^3^
Bottle weight data^7^, N, %^6^	5221	74.1^3^	4492	75.8^3^	3578	76.8^3^	2559	76.7^3^	1520	72.5^3^	714	68.1^3^	239	63.4^3^
100–111 g	2286	43.6	477	10.6	102	2.9	35	1.3	18	1.1	17	2.2	4	1.1
90–99 g	2168	41.4	888	19.7	279	7.8	61	2.4	16	1.0	6	0.8	0	0.0
80–89 g	529	10.1	1711	38.0	628	17.5	223	8.6	62	3.9	20	2.6	7	2.7
70–79 g	123	2.3	956	21.3	941	26.2	368	14.3	130	8.2	38	4.8	7	2.7
60–69 g	63	1.2	296	6.6	970	27.0	585	22.7	237	15.0	66	8.4	21	8.0
50–59 g	25	0.5	92	2.0	452	12.6	675	26.2	300	19.0	132	16.8	36	13.6
40–49 g	18	0.3	42	0.9	139	3.9	388	15.0	370	23.4	163	20.8	60	22.7
30–39 g	9	0.2	27	0.6	51	1.4	194	7.5	292	18.5	187	23.9	68	25.8
26–29 g	0	0.0	3	0.1	16	0.4	30	1.2	95	6.0	85	10.8	36	13.6
No bottle, N, %^6^	1801	25.6^3^	1427	24.1^3^	1064	22.8^3^	757	22.7^3^	516	24.6^3^	264	25.2^3^	113	30.0^3^
Recall + bottle weight data, N, %^6^	5103	72.5^3^	4133	69.8^3^	3173	68.1^3^	2223	66.6^3^	1249	59.6^3^	507	48.4^3^	145	38.5^3^

1 Included pregnant women whose Hb at registration was >10 g/L and were thus eligible for daily multiple micronutrient supplement (MMS) prophylaxis.

2 Excluded n = 7 and 2 women at first and second follow-up visits, respectively, without gestational week at registration.

3 Numbers are those mentioned in the Results.

4 Excluded n = 362, 160, 88, 40, 51, 13, and 7 women at first, second, third, fourth, fifth, sixth, and seventh follow-up, respectively, due to antenatal visits conducted without seeing midwives.

5 Excluded n = 124, 122, 112, 80, 53, 27, and 17 women at first, second third, fourth, fifth, sixth and seventh follow-up visits, respectively, due to multiple data entry errors or don’t know responses.

6 % refers to the percentage women eligible for adherence assessment.

7 Excluded n = 20 (0.3), 6 (0.1), 59 (1.3), 22 (0.7), 60 (2.9), 70 (6.7), and 25 (6.6) women at first, second, third, fourth, fifth, sixth, and seventh follow-up visit, respectively, with out-of-range MMS bottle weights (<26 g or >111 g, representing cutoffs for empty and full bottles, respectively).

We compared groups of women who failed to bring back bottles (noncompliers) to those who did (compliers) at follow-up visits on general characteristics, recalled MMS tablets missed, and habituality in compliance. At a given visit, 22.8%–32.1% of women were noncompliant (Supplementary Table 3). Age distributions were comparable, but noncompliers were slightly less well educated (evident at 6 of 7 visits, *P* < 0.05), more likely to live in versus outside of camps (30.1%–44.3% vs 19.8%-24.6%) (*P* < 0.001) and less likely to be nulliparous (1.8%–8.7% vs 4.2%–14.1%) (*P* < 0.001).

Among recipients with ≥ 2 follow-up visits, bottle return behavior was admixed, illustrated by only 40.9% of women not returning their bottle at their second follow-up visit also not doing so at their first follow-up, and only 22.2% of noncompliers at the third visit having done so at both earlier visits. The percentage of noncompliers by the fifth and later follow-up visits who had never returned their bottle previously decreased to <10% (Table 3). Bottle returnees were more consistent in their behavior, with 80.9% decreasing to 44.0% at second through seventh visits, having returned their bottle at all previous visits (bolded values).

**TABLE 3:** Histories of noncompliant and compliant patterns of MMS bottle return by compliance at each follow-up visit, all trimester registrants, 13 MMS-assigned UNRWA clinics, Jordan, March to December 2023^1^.

No. of previous bottle-compliant visits	Bottle-return compliance at -																											
	First follow-up visit				Second follow-up visit				Third follow-up visit				Fourth follow-up visit				Fifth follow-up visit				Sixth follow-up visit				Seventh follow-up visit			
	Noncompliers (N = 1801, 100%)^2^		Compliers (N = 5241, 100%)^2^		Noncompliers (N = 1324, 73.5%)		Compliers (N = 4307, 82.2%)		Noncompliers (N = 963, 53.5%)		Compliers (N = 3371, 64.3%)		Noncompliers (N = 668, 37.1%)		Compliers (N = 2423, 46.2%)		Noncompliers (N = 354, 19.7%)		Compliers (N = 1203, 29.5%)		Noncompliers (N = 231, 12.8%)		Compliers (N = 713, 13.6%)		Noncompliers (N = 98, 5.4%)		Compliers (N = 236, 4.5%)	
	n	%	n	%	n	%	n	%	n	%	n	%	n	%	n	%	n	%	n	%	n	%	n	%	n	%	n	%
None	1801	100	---	---	540	40.8^3^	0	0.0	214	22.2^3^	0	0.0	74	11.1^3^	0	0.0	22	6.2	0	0.0	8	3.5^3^	0	0.0	2	2.3^3^	0	0.0
1 time	---	---	5221	100	784	59.2	824	19.1	343	35.6	179	5.3	130	19.5	75	3.1	45	12.7	10	0.8	22	9.5	4	0.6	7	7.9	0	0.0
2 times	---	---	---	---	---	---	3483	80.9^3^	4056	42.2	843	25.0	223	33.4	213	8.8	79	22.3	49	4.1	40	17.3	15	2.1	10	11.2	3	1.5
3 times	---	---	---	---	---	---	---	---	---	---	2349	69.7^3^	241	36.1	664	27.4	116	32.8	142	11.8	54	23.4	38	5.3	15	16.9	6	2.9
4 times	---	---	---	---	---	---	---	---	---	---	---	---	---	---	14,571	60.7^3^	92	26.0	346	28.8	58	25.1	98	13.7	22	24.7	15	7.3
5 times	---	---	---	---	---	---	---	---	---	---	---	---	---	---	---	---	---	---	656	54.5^3^	49	21.2	199	27.9	20	22.5	32	15.6
6 times	---	---	---	---	---	---	---	---	---	---	---	---	---	---	---	---	---	---	---	---	---	---	359	50.4^3^	13	14.6	59	28.8
7 times	---	---	---	---	---	---	---	---	---	---	---	---	---	---	---	---	---	---	---	---	---	---	---	---	---	---	90	43.9

1 Number of recipients by follow-up visit for this longitudinal analysis, which requires linking >1 follow-up visits per woman after registration with bottle return data, is far less than cross-sectional tallies of follow-up visits in Table 2, which are not dependent on numbers of follow-up visits.

2 Number of women across trimesters of registration with >1 follow-up visits with bottle return data. Sequential reductions in follow-up visit n (%) represent combined effects of pregnancy termination, missing data, and censoring at end-of-study.

3 Percentages under “Non-compliers” and “ Compliers” columns, provide estimates of habitual patterns of women, respectively, never and always returning with an MMS bottle at each follow-up visit.

Noncompliers more frequently recalled having missed supplement days during the first 5 intervals. Although statistically significant, differences were modest, with 73.5%–83.5% of women in both groups having missed <2 d, and 0%–5.3% doing so for >30 d in any 1 interval (Table 4). Differences in recalled tablet use between noncompliers and compliers were unaffected by education, camp residence, and parity (data not shown), such that if biases existed in the validation analyses as a result of bottle noncompliance, they were likely minor.

**TABLE 4:** Recalled days missed taking multiple micronutrient supplements (MMS)^1^ during previous intervals at first to seventh follow-up visits among recipients by compliance with instructions to return with bottle, all trimester registrants, 13 MMS-assigned UNRWA clinics, Jordan, March to December 2023.

Recalled days missed, N, %^3^	Follow-up antenatal care visit number																											
	First follow-up visit^2^				Second follow-up visit^2^				Third follow-up visit^2^				Fourth follow-up visit^2^				Fifth follow-up visit^2^				Sixth follow-up visit				Seventh follow-up visit			
	Noncompliers		Compliers		Noncompliers		Compliers		Noncompliers		Compliers		Noncompliers		Compliers		Noncompliers		Compliers		Noncompliers		Compliers		Noncompliers		Compliers	
	n	%	n	%	n	%	n	%	n	%	n	%	n	%	n	%	n	%	n	%	n	%	n	%	n	%	n	%
	1774	100.0	5125	100.0	1392	100.0	4405	100.0	1033	100.0	3497	100.0	732	100.0	2504	100.0	492	100.0	1491	100.0	251	100.0	702	100.0	104	100.0	231	100.0
0–2	1434	80.8^4^	3950	77.1^4^	1038	74.6^4^	3341	75.8^4^	759	73.5^4^	2688	76.9^4^	540	73.8^4^	1969	78.6^4^	375	76.2^4^	1184	79.4^4^	195	77.7^4^	567	80.8^4^	86	82.7^4^	193	83.5^4^
3–4	94	5.3	479	9.3	99	7.1	456	10.4	65	6.3	388	11.1	49	6.7	224	8.9	24	4.9	129	8.7	18	7.2	56	8.0	5	4.8	15	6.5
5–9	92	5.2	378	7.4	97	7.0	350	7.9	69	6.7	254	7.3	51	7.0	188	7.5	40	8.1	100	6.7	15	6.0	28	4.0	4	3.8	13	5.6
10–29	99	5.6	245	4.8	101	7.3	200	4.5	85	8.2	123	3.5	61	8.3	98	3.9	34	6.9	58	3.9	19	7.6	36	5.1	4	3.8	10	4.3
30+	55	3.1^4^	73	1.4^4^	57	4.1^4^	58	1.3^4^	55	5.3^4^	44	1.3^4^	31	4.2^4^	25	1.0^4^	19	3.9^4^	20	1.3^4^	4	1.6^4^	15	2.1^4^	5	4.8^4^	0	0.0^4^

1 Included pregnant women whose Hb at registration was >10 g/L and were thus eligible for MMS prophylaxis.

2 *P* value <0.0001 by ꭓ^2^ test.

3 Excluded N = 27 and 97 women without bottle and with bottle at first follow-up, N = 35 and 87 women without bottle and with bottle at second follow-up, N = 31 and 81 women without bottle and with bottle at third follow-up, N = 25 and 55 women without bottle and with bottle at fourth follow-up, N = 24 and 29 women without bottle and with bottle at fifth follow-up, N = 13 and 12 women without bottle and with bottle at sixth follow-up, and N = 9 and 8 women without bottle and with bottle at seventh follow-up, respectively, due to data entry errors.

4 Numbers are those mentioned in the Results.

**FIGURE 2:**
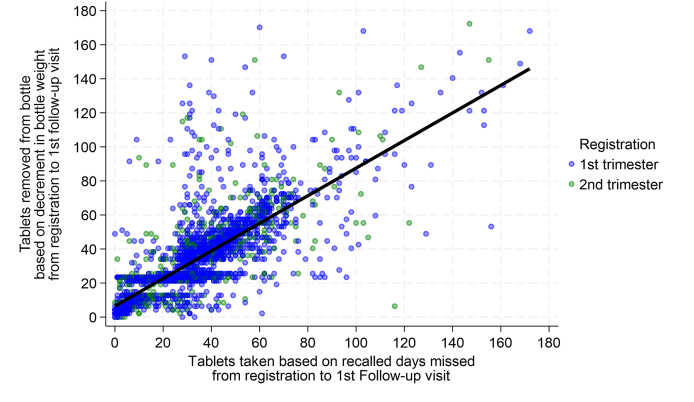
Joint distribution of tablets removed from bottles, based on decrement in bottle weight (y-axis), and tablets taken from bottles based on subtracting recalled days missed from interval length (x-axis) during the first follow-up interval among first (blue dots) and second (green dots) trimester registrants, 13 MMS UNRWA clinics, Jordan, March to December 2023. Simple linear regression model: N = 3117, Y = 6.43 + 0.81x (95% CI: 0.79, 0.83), r = 0.77 (see Table 5). MMS, multiple micronutrient supplementation; UNRWA, United Nations Relief and Works Agency for Palestine Refugees in the Near East.

Table 5 summarizes interval-specific, simple linear regression models representing the relationship of estimated tablets removed, based on decrement in bottle weight (y-axis), to reported tablets taken, based on recall (x-axis), for first and second trimester registrants combined, as models were similar by interval for both trimesters (Supplementary Table 4). Third trimester registrants, comprising 2.8% of all registrants (Table 1), were excluded from this analysis due to their late registration and truncated follow-up patterns nearing term. Across consecutive follow-up intervals of gradually smaller subsets of recipients from each previous visit, the slope (β_1_) consistently remained between 0.88 and 0.78 (Table 5), albeit with gradually widening 95% CIs, indicating fewer tablets being removed per decrement in bottle weight than calculated to have been taken by recall. Y-intercepts (β_0_) remained near the origin (range: 1.07–6.43), and correlation coefficients (r) varied from an initial high value of 0.77–0.65 to 0.56 over subsequent intervals. RMSEs, revealing an average error between predicted and actual numbers of tablets removed from bottles, varied from 10.3–12.4, representing 7%–10% of the range in tablets removed during each interval (Table 5). A scatterplot with superimposed regression line is illustrated for the first follow-up interval in Figure 2, with graphs for all intervals displayed in Supplementary Figures 1a–g.

**TABLE 5:** Follow-up interval-specific MMS tablets removed from bottles based on decrement in bottle weight^1^ regressed on tablets taken from bottles^2^ based on recalled days missed, first and second trimester combined, 13 MMS-assigned UNRWA clinics, Jordan, March to December 2023.

Follow-up interval	N	Tablets removed by decrement in bottle weight			Simple linear regression summaries					
		Mean	SD^3^	Range^4^	β_0_	β_1_	95% CI	r	RMSE	NRMSE
First	4977	31.58	19.53	172	6.43	0.81	(0.79−0.83)	0.77	12.4	0.07
Second	3248	30.76	15.67	142	5.04	0.79	(0.76−0.82)	0.65	11.8	0.08
Third	2716	29.54	14.61	155	2.65	0.85	(0.81−0.89)	0.62	11.5	0.07
Fourth	1974	28.67	13.97	129	2.37	0.86	(0.81−0.91)	0.56	11.5	0.09
Fifth	1184	26.61	13.14	106	3.08	0.84	(0.78−0.90)	0.59	10.6	0.10
Sixth	547	23.65	12.60	123	4.15	0.78	(0.69−0.87)	0.58	10.3	0.08
Seventh	184	21.52	13.98	117	1.07	0.88	(0.71−1.05)	0.60	11.2	0.10

β_0_, y intercept; β_1_, regression slope; 95% CI, 95% confidence interval for β_1_; r, Pearson correlation coefficient; RMSE, root mean square error (SD of residuals); NRMSE, normalized RMSE (RMSE / range).

1 Estimated tablets removed based on decrement in bottle weight (g) from previous to current follow-up visit divided by 0.47 g (tablet weight based on pilot study analysis).

2 Estimated tablets taken from bottle obtained by subtracting recalled days missed from number of days in a given follow-up interval.

3 Standard deviation.

4 Ranges represent differences between interval-specific minimum (least adherent, equal to 0 for each interval; data not shown) and maximum (longest, most adherent) values between any 2 sequential visits. All ranges are constrained by a maximum of 180 tablets in a bottle.

**FIGURE 3:**
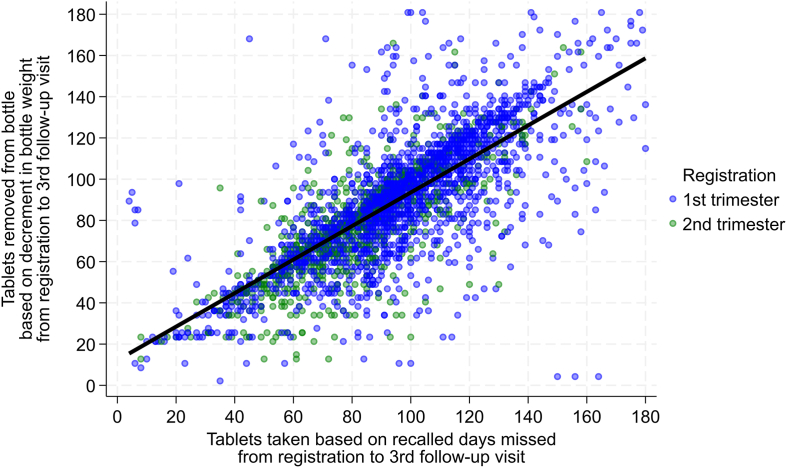
Cumulative joint distributions of tablets removed from bottles based on decrement in bottle weight (y-axis) at 0.47 g/tablet, and tablets taken from bottle based on subtracting recalled days missed from interval lengths (x-axis), summed across first 3 follow-up intervals from registration, representing a median (IQR) of 96 (78–114) days of prescribed MMS use and 24 (20–29) weeks of gestation (Table 2). Simple linear regression model: N = 3117, Y = 12.17 + 0.81x (95% CI: 0.78, 0.84), r = 0.76 (see Table 6). MMS, multiple micronutrient supplementation.

Regression models representing the cumulative averages of tablets removed as a function of cumulative tablets taken, per their respective estimation procedures, revealed slopes (β_1_) of 0.84–0.77, with initially narrow but then gradually widening 95% CIs and by correlations (r) of 0.79–0.64 (Table 6), which were comparable with interval-specific estimates. RMSE estimates increased from 15.5 after 2 intervals to ∼24 tablets by the fifth to seventh visits, reflecting 9%–13% of the ranges in tablets cumulatively removed through the 7 follow-up intervals. Similar cumulative regression coefficients were separately observed among first and second trimester registrants (Supplementary Table 5). The cumulative joint distribution with modeled regression line through the third follow-up visit is depicted in Figure 3, by which time women had been exposed to a median (IQR) of 96 (78–114) prescribed supplement days since registration by 24 (20–29) wk of gestation (Table 2). Graphs of cumulative distributions with regression lines at each follow-up visit are displayed in Supplementary Figure 2a–g.

**TABLE 6:** Cumulative MMS tablets removed from bottles based on decrement in bottle weight^1^ regressed on cumulative tablets taken from bottles based on recalled days missed^2^ between registration and each follow-up visit, first and second trimester registrants combined, 13 MMS-assigned UNRWA clinics, Jordan, March to December 2023.

Cumulative follow-up interval	N	Tablets removed by decrement in bottle weight			Simple linear regression summaries					
		Mean	SD^3^	Range^4^	β_0_	β_1_	95% CI	r	RMSE	NRMSE
First	4977	31.58	19.53	172	6.43	0.81	(0.79–0.83)	0.77	12.4	0.07
Second	4041	60.00	25.18	174	8.97	0.82	(0.80–0.84)	0.79	15.5	0.09
Third	3117	86.51	28.66	178	12.17	0.81	(0.78–0.84)	0.76	18.7	0.10
Fourth	2206	109.75	31.62	180	11.88	0.83	(0.80–0.86)	0.73	21.8	0.12
Fifth	1246	127.39	32.78	178	11.39	0.84	(0.79–0.89)	0.70	23.6	0.13
Sixth	507	136.94	31.94	178	23.09	0.77	(0.69–0.85)	0.66	24.1	0.13
Seventh	145	140.37	30.37	174	21.45	0.79	(0.64–0.94)	0.64	23.4	0.13

b_0_, y intercept; b_1_, regression slope; 95% CI, 95% confidence interval for b_1_; r, Pearson correlation coefficient; RMSE, root mean square error (SD of residuals); NRMSE, normalized RMSE (RMSE / range).

1 Cumulative tablets removed from bottle obtained by subtracting bottle weight (g) at the current follow-up visit from assigned bottle weight at registration. (111 g comprising weight of bottle, 180 tablets at 0.47 g each (tablet weight based on pilot study analysis), and desiccant canister at 2 g each).

2 Cumulative estimation of tablets taken from bottle obtained by subtracting the sum of recalled days missed up to the time of each follow-up visit from 180 (initial tablets in bottle at registration).

3 Standard deviation.

4 Ranges represent differences between minimum (least adherent) and maximum (longest duration and most adherent) values for cumulative distributions at each follow-up visit. All ranges are constrained by a maximum of 180 tablets in a bottle.

## Discussion

Randomized trials have revealed antenatal MMS to reduce risks of adverse pregnancy outcomes [15], leading WHO to issue in 2020 a contextual recommendation that antenatal micronutrient supplements be deployed accompanied by rigorous implementation research [17]. High adherence is a key MMS implementation indicator that, when combined with early introduction in pregnancy, may improve birth weight relative to lower adherence and later introduction [26]. Yet, although excellent methodological resources exist to guide scaling up and monitoring MMS delivery [24], studies that have prospectively validated methods to assess MMS adherence remain lacking.

This validation study of one approach to assess maternal adherence to daily MMS was nested into an implementation trial that compared performance of prophylactic MMS with a pre-existing IFA supplement regimen in a health care system annually serving ∼20,000 pregnant women [25,27,29]. During the pilot phase, midwives observed that women could more readily recall tablet days missed than taken, guiding the construction of our adherence indicator. As MMS was issued in 180-count bottles at registration, with a known weight of each full bottle and tablet, we posited that decrements in bottle weight, assessed by midwives on digital scales, would quantitatively align with tablets taken based on recall.

In this setting, across consecutive intervals, linear regression analyses revealed slopes (β_1_) of ∼0.83, indicating that, on average, for every 10 tablets estimated consumed, obtained by subtracting recalled missed days from interval length, 8–9 were removed based on change in bottle weight. The narrowly distributed slopes and 95% CIs offered evidence of a reliably precise method of estimating average, daily MMS tablet removal from bottles throughout pregnancy with this recall-based method. The ∼17% average underestimate of tablets removed, or ∼20% overestimate of tablets taken, suggests that recipients recalled too few missed days. This may possibly reflect limited time for thoughtful recall during brief and busy antenatal care visits following moderately variable follow-up intervals, or a desire for responses to align more closely with prescriptive use. Alternatively, a β_1_ <1 as repeatedly observed may also reflect, in part, attenuation bias associated with random measurement error in the sole predictor variable, tablet intake that has been based on recalled days missed [30]. Model precision at the individual level was apparent from the estimated RMSE, which across individual intervals indicated an average error of 10–12 tablets in predicting tablets removed. When normalized, RMSE accounted for 7%–10% of the measurement range. Model estimates for cumulative tablet use were comparable with those for individual intervals, except for a rising RMSE that paralleled an increasing standard deviation over time, though remaining roughly proportional to the range. As another indicator of model fit, follow-up interval coefficients of determination (R^2^) of ∼0.40 and ∼0.50, obtained by squaring Pearson correlation coefficients in TABLE 5, TABLE 6, revealed 50%–60% unexplained variation, suggesting caution in applying correction factors based on the current model to adjust individual recall-based estimates of adherence.

We have found no similarly designed validation studies of MMS adherence to which our findings can be compared. Although momentum is growing to implement MMS [5,24,[31], [32], [33]], most antenatal care systems in lower-income countries still deliver iron-folic acid supplements per WHO policy [34]. Indicators of coverage and, at times, adherence have been explored by analyzing nationally representative household survey data on maternal recall of numbers of IFA or other iron supplements received or consumed during the most recent pregnancy within the previous 2–5 y [33], prompting concerns about validity of such lengthy postpartum recall periods [[35], [36], [37]]. In Nepal, accuracy of self-reported tablet receipt, compared with independent documentation of issuance, declined 1–2 y after delivery [37], whereas another study designed to validate 2016 Nepal Demographic Health Survey questions on maternal IFA receipt, asked at ∼6 mo postpartum, revealed systematic patterns of both under- and over-reporting of tablet receipt [38]. These experiences emphasize the value of prospectively validating in-system MMS coverage and adherence indicators. A recent evaluation of a district antenatal MMS program launched in Indonesia adopted the Ministry of Health’s IFA goal of ≥90-d of supplement receipt to define adequate MMS adherence, which was met by 75% of pregnant women [39], although data on validity were not reported.

The current study had several novel features, strengths, limitations, and lessons learned that have been adapted to assist in evaluating MMS in other countries [22,24,40,41]. Anticipated gradual progress in establishing a new MMS program prompted a process of prospectively noting and weighting the likely importance of events, including the pilot phase, in advancing toward an MMS launch within the UNRWA health system, leading to an “enablement graph” [22], which is being adopted elsewhere (C. Ajello, personal communication, August 2025). Assessment of supplement adherence by asking women to recall days missed emerged from midwives’ observations during the pilot phase. The study involved a strict 10-mo period of registration and follow-up, with censoring of later data for analysis in a refugee health care system where the vast majority of women complete 7 to ∼8 antenatal visits [25,27,29]. This resulted in a continuous, unselected subsetting of women attending later visits, evident across all visits by ∼95% of follow-ups being attended by midwives, ∼95% of women reporting having missed ≥2 d of MMS, ∼75% of women consistently returning with MMS bottles, and regression coefficients and normalized RMSEs that remained stable over the sequence of visits. Women who returned bottles were more habitual in doing so, whereas noncompliers were admixed in their behavior. Although in this study only minor demographic and adherence differences were observed between noncompliers and compliers, this may be an important factor for other studies to surveil in future studies.

Validation of maternal reports hinged on an assumption that reductions in bottle weight would result from MMS tablets being consumed absent bias in recalling days missed. This expectation proved reasonably correct, on average, evident in a repeatedly observed, strong bivariate linear relationship between tablets “removed” and “taken” from bottles. An important assumption, however, is that bottle weighing and recalling missed days during clinic visits were independent activities. Midwives were guided to keep the 2 activities as “separate” as possible. In the course of health-related discussions, bottles were weighed and recorded, without inviting or dissuading discussion about the activity. If asked for a reason bottles were being weighed, midwives advised they were checking on tablet supply. However, although not a raised concern during reviews with midwives, bottle weighing may have prompted some under-reporting of days missed, which could in part explain overestimated tablet intake. A factor possibly also contributing to reporting bias could be a typically short, uncomplicated patient visit, often lasting < 5 min, limiting time for a more structured probe to improve recall accuracy. Recall may have also become less accurate for longer follow-up intervals. The possibility of sharing supplements was not investigated, which is plausible given that “multivitamins” are a highly valued, costly product when purchased in markets [27], meriting future investigation that may improve adherence assessment. Extending inquiries, however, requires remaining sensitive to patient-midwife interaction time in busy clinic settings, and resources needed to develop, train, execute, and analyze data from expanded protocols in limited resource, service-directed systems.

Although this study was part of a systems trial that coevaluated IFA supplement use [25,27,29], in the UNRWA system, 1–6 blister packs of 10-count IFA supplements have conventionally been prescribed as a standard-of-care with instructions to take tablets 2, 3, or 7 times a week, per clinical judgment. Under these conditions, we found it impractical to design and implement a comparable validation study of recalled IFA use, leaving this study to focus on MMS.

Validated adherence is a key contextual outcome to define, quantify, and guide the incorporation of MMS into antenatal health care. Maternal recall of days missed, combined with dated follow-up visits needed to generate interval length, offered in this health care setting, a reliable and reasonably valid approach to assess antenatal adherence to MMS use when tablets are issued in single, high-volume bottles.

## Author contributions

The authors’ responsibilities were as follows – MH, RH, TA, AS, LA, KPW, KMH, and KK designed the research; MH, LA, RH, TA, and AS conducted the research; MH and SA analyzed the data; MH and KPW cowrote the article, which was reviewed and edited by KMH and KK. MH has responsibility for the final content; and all authors: read and approved the final manuscript.

## Data availability

The datasets used and/or analyzed during the current study are available from the corresponding author on reasonable request.

## Funding

This study was funded by the Vitamin Angels Alliance, Goleta, CA, USA. UNIMMAP-formulated Multiple Micronutrient Supplements were donated by the Kirk Humanitarian Foundation, Salt Lake City, UT, United States. Technical support was provided by Program in Human Nutrition at Johns Hopkins Bloomberg School of Public Health, Baltimore, MD, United States, and Sight and Life Foundation, Kaiseraugst, Switzerland.

## Conflict of interests

KH declares her part-time position as the Vice President for Nutrition at the Vitamin Angels Alliance. The other authors have nothing to declare.
