# Supplementary material for: Validation Study of Adherence to Daily Multiple Micronutrient Supplementation by Palestine Refugee Women Attending United nations Relief and Works Agency for Palestine Refugees in the Near East Health Clinics in Jordan
Source: Curr Dev Nutr. 2025 Oct 30;9(12):107588. doi: 10.1016/j.cdnut.2025.107588 (PMC12721268; doi:10.1016/j.cdnut.2025.107588)
Supplement: multimedia component 1 [file mmc1.docx]

**Validation Study of Adherence to Daily Multiple Micronutrient Supplementation by Palestine Refugee Women Attending UNRWA Health Clinics in Jordan, Masako Horino**

**Supplementary Table 1. UNIMMAP^1^ formulation of multiple
micronutrient supplements (MMS) dispensed in 180-count bottles
for implementation research, UNRWA MMS-assigned clinics,
Jordan, March to December 2023**

| Nutrient | Chemical entity | UNIMMAP MMS^1^ |
| --- | --- | --- |
| Iron | Ferrous fumarate | 30 mg |
| Folic acid | Folic acid | 400 mcg |
| Vitamin A | Retinyl acetate | 800 mcg RAE^2^ |
| Vitamin D | Cholecalciferol | 5 mcg (200 IU) |
| Vitamin E | Alpha tocopheryl succinate | 10 mg α-TE^3^ |
| Vitamin C | L-Ascorbic acid | 70 mg |
| Vitamin B_1_ | Thiamine mononitrate | 1.4 mg |
| Vitamin B_2_ | Riboflavin | 1.4 mg |
| Vitamin B_3_ | Niacinamide | 18 mg NE^4^ |
| Vitamin B_6_ | Pyridoxine hydrochloride | 1.9 mg |
| Vitamin B_12_ | Cyanocobalamin | 2.6 mcg |
| Iodine | Potassium iodine | 150 mcg |
| Zinc | Zinc oxide | 15 mg |
| Selenium | Sodium selenite | 65 mcg |
| Copper | Cupric oxide | 2 mg |

^1^ UNIMMAP MMS = United Nations International Multiple Micronutrient Antenatal Preparation^1^

^2^ RAE = Retinol activity equivalents

^3^ α-TE = alpha-tocopherol equivalents

^4^ NE = Niacin equivalents

**References**

1. WHO Model List of Essential Medicines – 22nd List, 2021. World Health Organization, Geneva. 2021. Accessed February 23, 2025. <https://www.who.int/publications/i/item/WHO-MHP-HPS-EML-2021.02>.

**Supplementary Table 2. Distribution of women eligible for multiple micronutrient supplementation (MMS) prophylaxis^1^ by month and trimester of registration, 13 MMS-assigned UNRWA^2^ clinics, Jordan, March to December 2023**

|  | **1st trimester** | | **2nd trimester** | | **3rd trimester** | | **All registrants^3^** | |
| --- | --- | --- | --- | --- | --- | --- | --- | --- |
| **Month of registration** | n | % | n | % | n | % | n | % |
| Total | 6617 | 100.0 | 2826 | 100 | 271 | 100 | 9754 | 100 |
| March | 698 | 10.5 | 300 | 10.6 | 27 | 10.0 | 1027 | **10.5** |
| April | 404 | 6.1 | 156 | 5.5 | 14 | 5.2 | 576 | **5.9** |
| May | 875 | 13.2 | 468 | 16.6 | 40 | 14.8 | 1385 | **14.2** |
| June | 595 | 9.0 | 233 | 8.2 | 28 | 10.3 | 863 | **8.8** |
| July | 743 | 11.2 | 316 | 11.2 | 30 | 11.1 | 1093 | **11.2** |
| August | 664 | 10.0 | 322 | 11.4 | 35 | 12.9 | 1023 | **10.5** |
| September | 678 | 10.2 | 300 | 10.6 | 33 | 12.2 | 1011 | **10.4** |
| October | 681 | 10.3 | 265 | 9.4 | 17 | 6.3 | 967 | **9.9** |
| November | 629 | 9.5 | 219 | 7.7 | 25 | 9.2 | 880 | **9.0** |
| December | 650 | 9.8 | 247 | 8.7 | 22 | 8.1 | 929 | **9.5** |

^1^ Included pregnant women whose Hb at registration was ≥10 g/L and thus eligible for MMS prophylaxis.

^2^ UNRWA = United Nations Relief and Works Agency for Palestine Refugees in the Near East

^3^ N=40 women with unknown trimester at registration.

Note: Bolded numbers are those mentioned in Results text.

**Supplementary Table 3. Characteristics of non-compliers and compliers with respect to bringing multiple micronutrient supplementation (MMS) bottles^1^ at 1^st^-7^th^ follow-up visits, all trimester registrants, 13 MMS-assigned UNRWA^2^ clinics, Jordan, March to December 2023.**

|  | **1st Follow-up Visit** | | **2nd Follow-up Visit** | | **3rd Follow-up Visit** | | **4th Follow-up Visit** | | **5th Follow-up Visit** | | **6^th^ Follow-up Visit** | | **7^th^ Follow-up Visit** | |
| --- | --- | --- | --- | --- | --- | --- | --- | --- | --- | --- | --- | --- | --- | --- |
| **Characteristic** | **Non-complier** | **Complier** | **Non-Complier** | **Complier** | **Non-Complier** | **Complier** | **Non- Complier** | **Complier** | **Non- Complier** | **Complier** | **Non-Complier** | **Complier** | **Non- Complier** | **Complier** |
|  | n (%) | n (%) | n (%) | n (%) | n (%) | n (%) | n (%) | n (%) | n (%) | n (%) | n (%) | n (%) | n (%) | n (%) |
| Women | 1801 (**25.7**) | 5221 (74.4) | 1427 (**24.1**) | 4492 (75.9) | 1064 (**22.9**) | 3578 (77.1) | 757 (**22.8**) | 2559 (77.2) | 516 (**25.3**) | 1520 (74.7) | 264 (**27.0**) | 714 (73.0) | 113 (**32.1**) | 239 (67.9) |
| Gestational week,  median (IQR) | 15 (11–21) | 14 (11–20) | 20 (16–26) | 19 (16–24) | 25 (21–31) | 24 (20–29) | 28 (25–33) | 28 (24–32) | 32 (29–35) | 31 (28–34) | 34 (32–36) | 33 (30–35) | 36 (32–38) | 33 (30–36) |
| Age at registration, years | 1801 | 5221 | 1427 | 4492* | 1064 | 3578 | 757 | 2559 | 516 | 1520 | 264 | 714 | 113 | 239 |
| 15-19 | 138 (7.7) | 414 (7.9) | 117 (8.2) | 329 (7.3) | 73 (6.9) | 257 (7.2) | 56 (7.4) | 181 (7.1) | 37 (7.2) | 118 (7.8) | 26 (9.9) | 50 (7.0) | 8 (7.1) | 19 (8.0) |
| 20-29 | 1060 (58.9) | 2970 (56.9) | 842 (59.0) | 2497 (55.6) | 632 (59.4) | 1961 (54.8) | 410 (54.2) | 1403 (54.8) | 290 (56.2) | 784 (51.6) | 131 (49.6) | 353 (49.4) | 59 (52.2) | 102 (42.7) |
| 30-39 | 544 (30.2) | 1617 (31.0) | 409 (28.7) | 1482 (33.0) | 312 (29.3) | 1204 (33.7) | 250 (33.0) | 865 (33.8) | 173 (33.5) | 545 (35.9) | 93 (35.2) | 279 (39.1) | 40 (35.4) | 104 (43.5) |
| 40-49 | 59 (3.3) | 220 (4.2) | 59 (4.1) | 184 (4.1) | 47 (4.4) | 156 (4.4) | 41 (5.4) | 110 (4.3) | 16 (3.1) | 73 (4.8) | 14 (5.3) | 32 (4.5) | 6 (5.3) | 14 (5.9) |
| Education^3^, N | 1797 | 5212* | 1421 | 4487* | 1061 | 3573** | 753 | 2555** | 514 | 1518 | 263 | 711* | 111 | 239* |
| Grades 1-10 | 888 (49.4) | 2355 (45.2) | 704 (49.5) | 2033 (45.3) | 539 (50.8) | 1609 (45.0) | 397 (52.7) | 1152 (45.1) | 259 (50.4) | 719 (47.4) | 145 (55.1) | 339 (47.7) | 66 (59.5) | 108 (45.2) |
| Grades 11-12 | 620 (34.5) | 1970 (**37.8**) | 496 (34.9) | 1687 (**37.6**) | 347 (32.7) | 1393 (**39.0**) | 257 (34.1) | 978 (**38.3**) | 183 (35.6) | 561 (37.0) | 92 (35.0) | 260 (**36.6**) | 35 (31.5) | 91 (**38.1**) |
| Some college or higher | 289 (16.1) | 887 (**17.0**) | 221 (15.6) | 767 (**17.1**) | 175 (16.5) | 571 (**16.0**) | 99 (13.2) | 425 (**16.6**) | 72 (14.0) | 238 (15.7) | 26 (9.9) | 112 (**15.8**) | 10 (9.0) | 40 (**16.7**) |
| Residence^4^, N | 1680 | 5025** | 1321 | 4284** | 989 | 3411** | 714 | 2421** | 481 | 1454** | 246 | 683** | 106 | 232** |
| Inside camp | 529 (**30.9**) | 1024 (**20.4**) | 428 (**32.4**) | 850 (**19.8**) | 298 (**30.1**) | 704 (**20.6**) | 231 (**32.4**) | 481 (**19.9**) | 159 (**33.1**) | 305 (**21.0**) | 103 (**41.9**) | 149 (**21.8**) | 47 (**44.3**) | 57 (**24.6**) |
| Outside camp | 1151 (67.3) | 3924 (78.1) | 893 (67.6) | 3434 (80.2) | 691 (69.9) | 2709 (79.4) | 483 (67.6) | 1940 (80.1) | 322 (66.9) | 1149 (79.0) | 143 (58.1) | 534 (78.2) | 59 (55.7) | 175 (75.4) |
| Parity, N | 1801 | 5221** | 1427 | 4492** | 1064 | 3578** | 757 | 2559** | 516 | 1520* | 264 | 714 | 113 | 239 |
| 0 | 156 (**8.7**) | 734 (**14.1**) | 105 (**7.4**) | 557 (**12.4**) | 73 (**6.9**) | 366 (**10.2**) | 39 (**5.2**) | 201 (**7.9**) | 24 (**4.7**) | 95 (**6.3**) | 8 (**3.0**) | 34 (**4.8**) | 2 (**1.8**) | 10 (**4.2**) |
| 1 | 441 (24.5) | 1309 (25.1) | 353 (24.7) | 1126 (25.1) | 234 (22.0) | 913 (25.5) | 159 (21.0) | 672 (26.3) | 99 (19.2) | 381 (25.1) | 60 (22.7) | 172 (24.1) | 26 (23.0) | 51 (21.3) |
| 2-4 | 938 (52.1) | 2575 (49.3) | 748 (52.4) | 2244 (50.0) | 585 (55.0) | 1812 (50.6) | 417 (55.1) | 1334 (52.1) | 306 (59.3) | 805 (53.0) | 145 (54.9) | 384 (53.8) | 57 (50.4) | 129 (54.0) |
| 5 or more | 266 (14.7) | 603 (11.6) | 221 (15.5) | 565 (12.6) | 172 (16.2) | 487 (13.6) | 142 (18.8) | 352 (13.8) | 87 (16.9) | 239 (15.7) | 51 (19.3) | 124 (17.4) | 28 (24.8) | 49 (20.5) |

^1^ Included pregnant women whose Hb at registration was >10 g/L and thus eligible for MMS prophylaxis .

^2^ UNRWA = United Nations Relief and Works Agency for Palestine Refugees in the Near East

^3^ Due to unknown education level, excluded N=4 women with no bottle and N=9 women with a bottle at 1^st^ follow-up visit, N=6 women with no bottle and N=5 with a bottle at 2^nd^ follow-up visit, N=3 women with no bottle and N=5 women with a bottle at 3^rd^ follow-up visit, N=4 women with no bottle and N=4 women with no bottle at 4^th^ follow-up visit, N=2 women with no bottle and N=2 woman with a bottle at 5^th^ follow-up visit, N=1 woman with no bottle and N=3 woman with a bottle at 6^th^ follow-up visit, and N=2 women with no bottle at 7^th^ follow-up visit.

^4^ Excluded N=121 women with no bottle (N=31 living abroad and N=90 unknown) and N=275 women with a bottle (N=77 living abroad and N=196 unknown) at 1^st^ follow-up visit, N=106 women with no bottle (N=26 living abroad and N=80 unknown) and N=208 women with a bottle (N=61 living abroad and N=147 unknown) at 2^nd^ follow-up visit, N=75 women with no bottle (N=20 living abroad and N=55 unknown) and N=167 women with a bottle (N=45 living abroad and N=122 unknown) at 3^rd^ follow-up visit, N=43 women with no bottle (N=10 living abroad and N=33 unknown) and N=138 women with a bottle (N=39 living abroad and N=99) at 4^th^ follow-up visit, and N=35 women with no bottle (N=10 living abroad and N=25 unknown) and N=66 women with a bottle (N=18 living abroad and N=48 unknown) at 5^th^ follow-up visit, N=18 women with no bottle (N=5 living abroad and N=13 unknown) and N=31 women with a bottle (N=10 living abroad and N=21 unknown) at 6^th^ follow-up visit, and N=11 women with no bottle (N=4 living abroad and N=7 unknown) and N=8 women with a bottle and unknown residence at 7^th^ follow-up visit.

* P-value <0.05 by ꭓ^2^ test; ** P-value <0.001 by ꭓ^2^ test

Note: Bolded numbers are those mentioned in Results text.**Supplementary Table 4. Interval specific multiple micronutrient supplementation (MMS) tablets removed based on decrement in bottle weight**^1^ **regressed on tablets recalled taken from a bottle**^2^ **over sequential follow-up intervals, among 1^st^ and 2^nd^ trimester registrants, 13 MMS-assigned UNRWA^3^ clinics, Jordan, March to December 2023.**

|  | **1st trimester registrants** | | | | | **2nd trimester registrants** | | | | |
| --- | --- | --- | --- | --- | --- | --- | --- | --- | --- | --- |
| **Follow-up Interval** | **N** | **b_0_** | **b_1_** | **95% CI** | **Pearson's correlation coefficient** | **N** | **b_0_** | **b_1_** | **95% CI** | **Pearson's correlation coefficient** |
| First | 3491 | 6.51 | 0.81 | (0.79-0.83) | 0.76 | 1486 | 6.38 | 0.8 | (0.77-0.83) | 0.78 |
| Second | 2291 | 6.49 | 0.75 | (0.71-0.79) | 0.65 | 957 | 1.29 | 0.9 | (0.84-0.96) | 0.67 |
| Third | 1947 | 2.96 | 0.85 | (0.80-0.90) | 0.6 | 769 | 2.28 | 0.85 | (0.78-0.92) | 0.67 |
| Fourth | 1483 | 2.31 | 0.86 | (0.80-0.92) | 0.56 | 491 | 2.96 | 0.82 | (0.71-0.93) | 0.54 |
| Fifth | 949 | 3.31 | 0.83 | (0.75-0.91) | 0.57 | 235 | 3.63 | 0.78 | (0.64-0.92) | 0.58 |
| Sixth | 451 | 4.96 | 0.76 | (0.65-0.87) | 0.54 | 96 | 4.25 | 0.68 | (0.50-0.86) | 0.6 |
| Seventh | 157 | 2.02 | 0.85 | (0.71-1.05) | 0.54 | 27 | -1.06 | 0.93 | (0.75-1.11) | 0.90 |

^1^ Estimated tablets removed based on decrement in bottle weight (g) from previous to current follow-up visit

divided by 0.47 g (measured weight of each tablet)

^2^ Estimated tablets taken from bottle obtained by subtracting recalled days missed from number of days in

a given follow-up interval.

^3^ UNRWA = United Nations Relief and Works Agency for Palestine Refugees in the Near East

**Supplementary Table 5. Cumulative multiple micronutrient supplement (MMS) tablets removed based on decrement in bottle weight**^1^ **regressed on tablets recalled taken from a bottle**^2^ **at sequential follow-up visits among 1^st^ and 2^nd^ trimester registrants, 13 MMS-assigned UNRWA^3^ clinics, Jordan, March to December 2023**

|  | **1st trimester registrants** | | | | | **2nd trimester registrants** | | | | |
| --- | --- | --- | --- | --- | --- | --- | --- | --- | --- | --- |
| **Follow-up Visit** | **N** | **b_0_** | **b_1_** | **95% CI** | **Pearson's correlation coefficient** | **N** | **b_0_** | **b_1_** | **95% CI** | **Pearson's correlation coefficient** |
| First | 3491 | 6.51 | 0.81 | (0.79-0.83) | 0.76 | 1486 | 6.38 | 0.80 | (0.77-0.83) | 0.78 |
| Second | 2849 | 9.79 | 0.81 | (0.79-0.83) | 0.79 | 1192 | 7.13 | 0.85 | (0.81-0.89) | 0.78 |
| Third | 2236 | 15.00 | 0.79 | (0.76-0.82) | 0.74 | 881 | 6.86 | 0.86 | (0.81-0.91) | 0.78 |
| Fourth | 1650 | 14.34 | 0.81 | (0.77-0.85) | 0.70 | 556 | 6.44 | 0.87 | (0.80-0.94) | 0.74 |
| Fifth | 999 | 15.90 | 0.81 | (0.24-1.38) | 0.66 | 247 | -2.86 | 0.95 | (0.85-1.05) | 0.76 |
| Sixth | 411 | 30.17 | 0.73 | (0.64-0.82) | 0.63 | 96 | 3.52 | 0.90 | (0.72-1.08) | 0.71 |
| Seventh | 118 | 23.47 | 0.79 | (0.62-0.96) | 0.64 | 27 | 37.63 | 0.62 | (0.25-0.99) | 0.55 |

^1^ Cumulative tablets removed from bottle obtained by subtracting bottle weight (g) at a current follow-up visit from assigned bottle weight at registration

(111 g comprising weight of bottle, 180 tablets at 0.47 g each (measured weight of single tablet), and desiccant canister at 2 g each).

^2^ Cumulative estimation of tablets taken from bottle obtained by subtracting the sum of recalled days missed up to the time of each follow-up visit from

180 (initial tablets in bottle at registration).

^3^ UNRWA = United Nations Relief and Works Agency for Palestine Refugees in the Near East

**Supplementary Figure 1a-g**. Joint distribuitions of tablets removed from bottles, based on decrement in bottle weight (y-axis), and tablets taken from bottles based on subtracting recalled days missed from interval length (x-axis) during the 1^st^ through 7^th^ follow-up intervals among 1st and 2nd trimester registrants, 13 MMS^1^ UNRWA^2^ clinics, Jordan, March to December 2023. See Table 5 for follow-up interval regression summaries.

^1^ MMS = multiple micronutrient supplementation

^2^ UNRWA = United Nations Relief and Works Agency for Palestine Refugees in the Near East

**Supplementary Figure 1a. Registration to 1^st^ follow-up visit (1^st^ interval)**

**Supplementary Figure 1b. First to second follow-up visit (2^nd^ interval)**

**Supplementary Figure 1c. Second to third follow-up visit (3rd interval)**

**Supplementary Figure 1d. Third to fourth follow-up visit (4th interval)**

**Supplementary Figure 1e. Fourth to fifth follow-up visit (5th interval)**

**Supplementary Figure 1f. Fith to sixth follow-up visit(6^th^ interval)**

**Supplementary Figure 1g. Sixth to seventh follow-up visit (7th interval)**

**Supplementary Figure 2a-g.** Cumulative joint distributions of tablets removed from bottles based on decrement in bottle weight (y-axis) at 0.47 g/tablet, and tablets taken from bottle based on subtracting recalled days missed from interval lengths (x-axis), summed across 1^st^ three follow-up intervals from registration, 13 MMS UNRWA clinics, Jordan, March to December 2023. See Table 6 for cumulative regression summaries.

^1^ MMS = multiple micronutrient supplementation

^2^ UNRWA = United Nations Relief and Works Agency for Palestine Refugees in the Near East

**Supplementary Figure 2a. Registration to 1^st^ follow-up visit**

**Supplementary Figure 2b. Registration to second follow-up visit**

**Supplementary Figure 2c. Registration to third follow-up visit**

**Supplementary Figure 2d. Registration to fourth follow-up visit**

**Supplementary Figure 2e. Registration to fifth follow-up visit**

**Supplementary Figure 2f. Registration to sixth follow-up visit**

**Supplementary Figure 2g. Registration to seventh follow-up visit**
